# Safety evaluation of frequent application of microbubble-enhanced focused ultrasound blood-brain-barrier opening

**DOI:** 10.1038/s41598-018-35677-w

**Published:** 2018-12-07

**Authors:** Hong-Chieh Tsai, Chih-Hung Tsai, Wen-Shiang Chen, Claude Inserra, Kuo-Chen Wei, Hao-Li Liu

**Affiliations:** 1 Department of Neurosurgery, Chang Gung Memorial Hospital at Linkou, Taoyuan, 333 Taiwan; 2 Graduate Institute of Clinical Medical Sciences and School of Traditional Chinese Medicine, Chang Gung University, Taoyuan, 333 Taiwan; 3 Department of Electrical Engineering, Chang-Gung University, Taoyuan, 333 Taiwan; 4 Department of Physical Medicine & Rehabilitation, National Taiwan University Hospital, Taipei, 100 Taiwan; 5 LabTAU, INSERM, Centre Léon Bérard, Université Lyon 1, Univ Lyon, Lyon, F-69003 France

## Abstract

Focused ultrasound (FUS) with the presence of microbubbles induces blood brain barrier (BBB) opening in targeted areas and facilitates drug delivery. However, recent studies have indicated that FUS-BBB opening with excessive exposure levels may be associated with inflammatory response and cellular/tissue damage. Multiple weekly FUS exposures have been shown to be safe for human subjects. However the effect of more frequent FUS exposures is still unknown. This study examines whether frequent focused ultrasound blood brain barrier opening is associated with aggravated behavioral, histopathologic change or brain tissue damage. Two protocols of focused ultrasound blood brain barrier opening were devised using different microbubble doses (0.15 µl/kg and 0.4 µl/kg). Focused ultrasound exposure at a threshold level of BBB-opening, below-threshold level, or above level for intracerebral hemorrhage were delivered every 2 days. Animal behavioral and physiological changes were examined and recorded. Brain tissue was examined for hemorrhage and apoptosis. Results indicate that frequent exposure of excessive focused ultrasound (1.4 mechanical index) produced minor and short-term behavioral changes despite significant tissue damage, while frequent BBB opening with threshold or below-threshold FUS exposure (0.33–0.8 mechanical index) did not cause behavioral or histological change. Immunofluorescent examination of rat brain tissue indicated that excessive doses of microbubble administration induce an apparent cellular apoptotic response, which may be exacerbated by intracerebral hemorrhage. Experimental results suggest that frequent focused ultrasound blood brain barrier opening with sufficient ultrasound exposure level and a microbubble dose can be safe and pose minimal risk to brain tissue.

## Introduction

The blood brain barrier (BBB) poses significant impediments to the treatment of malignant glioma of the brain^1^. The blood brain barrier is a multicellular vascular structure that tightly controls the passage of molecules and ions between the bloodstream and the brain. By selectively preventing the diffusion of hydrophilic molecules and pathogens from entering the brain parenchyma, it maintains an environment that ensures synaptic transmission and neuronal function. However, it also prevents the diffusion of large-molecule neuropeptides and more than 98% of small molecules drugs from entering the brain^2^, and thus presents a major challenge for medical treatment of CNS diseases such as brain tumors.

Glioblastoma (GBM) accounts for half of all primary malignant brain tumors^3^ and causes highest premature mortality among all malignant tumors^4,5^. One major challenge in GBM treatment is its inevitable recurrence^6,7^ caused by tumor cells infiltrating into surrounding normal functioning brain, thus making total surgical resection impossible without causing neurological deficit. Radiation therapy has only modest effect on these cells, and the BBB prevents effective chemotherapy due to low BBB permeability of chemotherapeutic agents^8,9^. Furthermore, many experimental therapeutic modalities that work on GBM cells *in vitro* cannot penetrate the BBB, and thus have little effect *in vivo*^10^. Effective treatment of brain tumors requires the high frequency administration of chemotherapeutic agents. For example, current standard glioma chemotherapeutic protocol requires daily application of temozolomide (TMZ) for 5 consecutive days every 28 days.

Three different strategies have been developed to overcome the challenge posed by BBB in drug delivery: intra-arterial infusion of hyperosmolar solutions or vasoactive drugs, direct injection of drugs into target area thus bypassing the BBB, or encapsulating drugs in liposomes or nanoparticles to be delivered across the BBB^11^. The first approach induces a transient rise in intracranial pressure, causes nonselective opening of the BBB in the vascular territory, and exposes large volumes of brain tissue to potentially toxic substances^12^. The second approach requires surgical device implantation and is limited by parenchymal drug diffusion. The efficacy of the third approach is limited by nanoparticle toxicity and bio-distribution^13,14^. In recent years, focused ultrasound (FUS) opening of the BBB has emerged as a promising new technique for enhancing drug delivery into the brain^15^. By injecting microbubbles (MB) intravenously and simultaneously applying burst-type ultrasound to targeted brain tissue, we can induce site-specific transient BBB opening and enhance CNS drug delivery without causing significant adverse effects^16^. Glioma animal experiments have shown that adding FUS-induced BBB opening to TMZ chemotherapy increases drug tumor tissue concentration and improves patient survival^17,18^. Focused ultrasound BBB opening is currently being investigated in clinical trials as an adjunct to brain tumor treatment.

Despite the great potential of FUS-BBB opening, several recent studies have highlighted possible safety issues associated with such treatment. Other than BBB opening, some reports have demonstrated that FUS + MB induces transient tissue edema, neuronal function suppression^19^, astroglial scarring^20^, transient ischemia, intracerebral hemorrhage^21^, and sterile inflammation^22,23^. Furthermore, all of these studies investigated FUS applied either once only or repeated on a weekly/bi-weekly basis^24^. However current standard glioma chemotherapeutic protocol requires daily application of TMZ for 5 consecutive days^25^. Based on the fact that BBB opening induced by FUS lasts for only 2–3 hours^26^, and that temozolomide has a mean elimination half-life of approximately 1.9 hours^27^, FUS-BBB opening procedure should be given as frequently as possible during the five-day dosing period to maximize its effect. Repeated FUS-BBB opening has been shown to be safe in various animal models^20,24,28,29^. However some recent reports indicate that application of FUS capable of disrupting the BBB may cause cellular apoptosis or sterile inflammation in the target tissue^22,23^, raising concerns about the safety of such a treatment modality. Furthermore, these studies only performed single exposures, or were repeated weekly or biweekly to allow for tissue recovery or remodeling. Previous glioma animal experiments have shown that FUS-BBB opening every other day as an adjunct to TMZ chemotherapy increased drug concentrations in the target area, improved tumor growth inhibition, and increased overall survival^17,18^. The safety of such frequent and repeated BBB-openings is still unclear.

This study seeks to examine whether such frequent FUS-BBB opening induces behavioral or physiological changes or causes aggravated brain damage, and to validate the safety of such procedures.

## Materials and Methods

### FUS instrumentation and calibration

**Figure 1:**
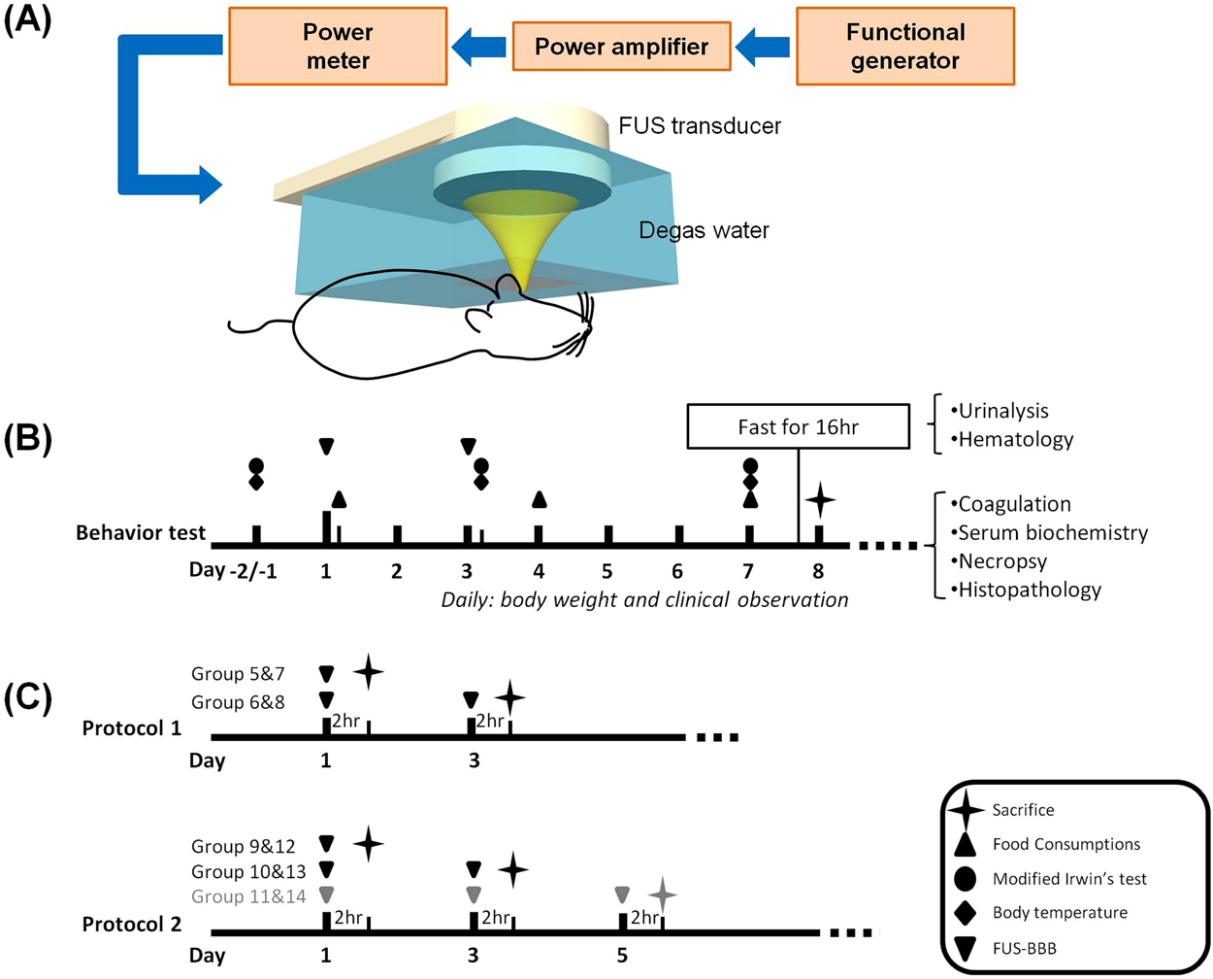
Experimental setup of focused ultrasound BBB opening and animal experimental design. (**A**) Focused ultrasound setup for blood-brain barrier opening, as described in the Materials and Methods section. (**B**) Experimental design for behavior test. Rats underwent FUS-BBB opening twice, followed by 7 days of observation before being sacrificed for necropsy. (**C**) Experimental design for effects of FUS-BBB opening on rat brain tissue. Rats were given FUS-BBB opening as designated, sacrificed 2 hours later and perfused, after which their tissue was harvested and examined.

The FUS instruments were described previously^19^. Briefly, the device is comprised of a function generator (Agilent, USA), a radiofrequency power amplifier (Amplifier Research, USA) and a focused ultrasound transducer (IMASONIC, France). A FUS frequency of 400 kHz was used. The acoustic pressure field was measured in a free field within an acrylic tank filled with deionized/degassed water by moving a calibrated needle hydrophone (HNA-0400, ONDA,USA) in steps of 0.22 mm, and the field of view in the axial and cross sections were respectively 10 × 90 mm and 10 × 10 mm (Fig. 1A). The diameter and length of the half-maximum pressure amplitude of the ultrasound field were respectively 2 and 15 mm. The measured acoustic pressure of 0, 0.2–0.3, 0.5 and 0.9 MPa corresponded to the mechanical index (MI) to 0.33–0.47, 0.8, and 1.4. The transcranial pressure measurement and distribution is shown in the Supplementary Fig. S1.

### FUS-induced BBB opening

The procedure for inducing BBB opening using focused ultrasound and IV injection of microbubbles was described previously^19^ (Fig. 1A). All animal procedures were approved by the Institutional Animal Care and Use Committee of Chang-Gung University and adhered to the experimental animal care guidelines. Briefly, male Sprague-Dawley rats weighing 311 ± 20 g were anesthetized with 3% isoflurane. The scalp was shaved with clippers followed by depilatory cream. Animals were placed in the prone position and fixed onto rodent stereotactic surgical frame, directly under an acrylic water tank that contained a 4 × 4 cm^2^ window sealed with a thin film of polyethylene membrane to allow the ultrasound to penetrate through its base. The method has been proven accurate in our previous studies^30^. The space between the skull and the thin-film window was filled with ultrasound gel. Lipid-shell Sulfur hexafluoride (SF6)-encapsulated microbubbles (SonoVue®, Bracco Suisse SA, Switzerland; 2–5 μm in mean diameter) and heparin (0.03 ml/kg; Agglutex, China Chemical and Pharmaceutical Corporation, Taiwan) were administered intravenously after dilution with normal saline solution to a total volume of 0.3 ml. Immediately after microbubble injection, ultrasound at the designated pressure was delivered over a total sonication duration of 120 seconds for single exposure to the left basal ganglion (coordinates from Bregma: AP, +2 mm; ML, 2 mm; 2 mm below skull). The acoustic exposure level of 0.33–0.47 MI, 0.8-MI, and 1.4-MI was delivered (duration = 120 s, burst length = 10 ms, burst repetition frequency = 1 Hz), with a repeated delivery cycle of day 1/3 for behavior test or day 1/3/5 for immunofluorescent staining.

With microbubble administration at 0.15 ml/kg, FUS exposure levels below 0.8-MI have been previously shown to be a threshold for consistent BBB opening without significant erythrocyte extravasations, while acoustic pressure of 1.4-MI causes significant intracerebral hemorrhage^16,18,19,26,31^. We therefore regard the former as “adequate FUS exposure”, while the later condition is regarded as “excessive FUS exposure”. To evaluate the effect of excessive microbubbles, we devised another FUS-BBB opening protocol using a microbubble dosage of 0.4 ml/kg, and measured its FUS-BBB opening threshold. We found that the BBB-opening threshold for the excessive MB group was 0.33-MI, while 0.8-MI caused significant intracerebral hemorrhage. Therefore we regard 0.4 ml/kg – 0.33-MI as “adequate FUS exposure” and 0.4 ml/kg – 0.8-MI as “excessive FUS exposure”.

### Animal experimental design for behavior test

To evaluate the effect of FUS-BBB opening on animal behaviors, male Sprague-Dawley rats were randomized and assigned to 4 groups according to the study design described below (Table 1, Fig. 1B). Focused-ultrasound opening of the BBB was performed on days 1 and 3, and the animals were sacrificed on day 8 for terminal necropsy. The following parameters were monitored: clinical observations, modified Irwin’s test^32,33^ plus body temperature, body weight, food consumption, hematology, serum biochemistry, coagulation, urinalysis, organ weights, macroscopic observations at necropsy, and microscopic examinations of collected tissues. In the modified Irwin’s test, the motor activity, behavioral changes, coordination, sensory/motor reflex responses and body temperature of test subjects were assessed before and after focused ultrasound treatment. The animal was observed and handled for 5 minutes in compliance with Irwin’s test setup, the number of occurrences of descriptive and categorical endpoints (as listed in the table) were counted and scored and averaged for the 10 animals in each group. To ensure objectivity and fairness, these experiments were carried out in QPS Taiwan, a third party GLP/GCP-compliant contract research organization for preclinical testing.

**Table 1:** Summary of experimental animal parameters.

Group	FUS strength	Exposure level (MI)	Dose volume of microbubble solution (mL/kg)	Animal number
1	None	0	—	10
2	Light	0.47	0.15	10
3	Moderate	0.8	0.15	10
4	Heavy	1.4	0.15	10

### Animal experimental design for tissue damage evaluation

To evaluate the effect of FUS-BBB opening on animal brain tissue, male Sprague-Dawley rats were randomized and assigned to 10 groups (group 5–14) according to the study design described below (Table 2, Fig. 1C). “Adequate FUS exposure” was defined as FUS application that produces consistent BBB opening without erythrocyte extravasation, while “excessive FUS exposure” was defined as FUS application that produces gross intracerebral hemorrhage. Focused-ultrasound opening of the BBB was performed on days 1, 3, and 5, and the animals were sacrificed 2 hours after the final FUS application. The rats were then perfused with 4% paraformaldehyde, after which their brains were harvested and sent for frozen section and immunofluorescent staining.

**Table 2:** Summary of the experimental parameters.

Group	Protocol	FUS strength	Exposure level (MI)	Dose volume of microbubble solution (mL/kg)	Exposure Frequency	Animal number
5	I	Moderate	0.8	0.15	Day 1	6
6	I	Moderate	0.8	0.15	Day 1, 3	6
7	I	Heavy	1.4	0.15	Day 1	6
8	I	Heavy	1.4	0.15	Day 1, 3	6
9	II	Moderate	0.33	0.4	Day 1	6
10	II	Moderate	0.33	0.4	Day 1, 3	6
11	II	Moderate	0.33	0.4	Day 1, 3, 5	6
12	II	Heavy	0.8	0.4	Day 1	6
13	II	Heavy	0.8	0.4	Day 1, 3	6
14	II	Heavy	0.8	0.4	Day 1, 3, 5	6

### Immunofluorescent staining and TUNEL assay

For immunofluorescent staining, male Sprague-Dawley rats were subjected to FUS-BBB treatment as described and sacrificed 2 hours after the last sonication session. EB dye was IV-injected as a bolus immediately after ultrasound exposure to serve as an indicator of BBB opening. The animals were anesthetized with isoflurane, and then euthanized with isoflurane overdose. They were then perfused transcardially with 0.9% normal saline, followed by 4% paraformaldehyde. The brains were harvested and put in sucrose for frozen sectioning. Frozen sections (thickness = 20 μm) were incubated for 1 hour in 0.3% BSA in PBST at room temperature. Anti-GFAP antibody (1:100; #AB5804, Millipore) was diluted in a blocking buffer and incubated at 4 °C overnight. The appropriate fluorophore-conjugated secondary antibody was added in a 1:200 dilution in the blocking buffer and incubated for 90 min at room temperature before mounting with DAPI (5 g/ml) and SlowFade Gold (Molecular Probes). Apoptosis in sections were assessed by the TUNEL assay using an *In situ* cell death detection kit according to manufacturer’s instructions (Roche). Immunofluorescent images were viewed with a fluorescence laser-scanning confocal microscope (Olympus FV10i, Center Valley, PA). The immunofluorescent image acquisition for the DAPI, Rhodamine and FITC channels were kept constant over all samples.

### Histopathalogical examination and analysis

Harvested brain samples were preserved in 10% neutral buffered formalin for 24 hours, washed with 70% ethanol, and embedded in paraffin using an embedding machine. From the paraffin blocks, 2 um thick serial sections were cut and stained using hematoxylin and eosin (H&E) dyes. Slices were observed under a light microscope.

### Statistical analysis

Individual data were tabulated and group means ± standard deviations were calculated using Microsoft Office Excel 2010. Statistical analysis was performed utilizing SigmaStat Statistical Software for Windows, Release 3.0 (Jandel Scientific Inc., USA). A significance level of 0.05 was used for all statistical tests. Summary statistics (sum, mean and standard deviation) of each group were calculated using Microsoft Excel 2010. In group comparison, homogeneity of data variance was assessed first by Equal Variance Test. A one-way analysis of variance (ANOVA) was applied to homogeneous data. Dunnett’s test was performed to compare each treatment group (Groups 2, 3 or 4) against the control group (Group 1) if the result of the one-way ANOVA showed statistically significant difference.

## Results

### Frequent and repetitive high FUS exposure causes short-term hypoactivity and ataxia

**Figure 2:**
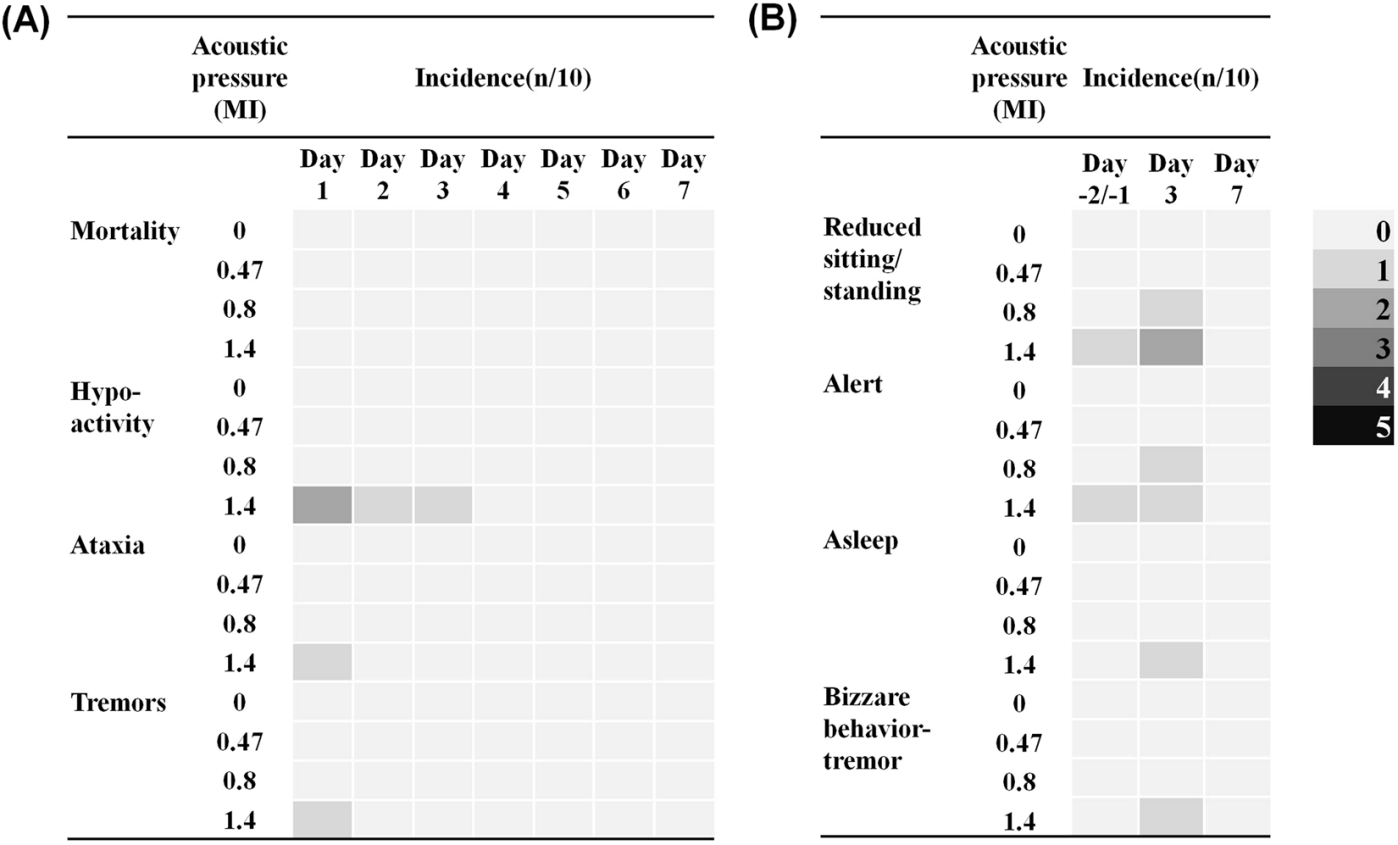
Summary of clinical observations and descriptive endpoint. (**A**) Summary of mouse mortality and clinical observations (n = 10). One out of 10 in the 1.4-MI exposure group displayed ataxia and limb tremor. Two out of 10 on Day 1 and 1 out of 10 on Days 2/3 in the 1.4-MI exposure group displayed hypoactivity. (**B**) Summary of descriptive endpoint (n = 10). One out of 10 in the 1.4-MI exposure group exhibited alert posturing, while one exhibited asleep posturing, and two displayed reduced sitting and standing on Day 3. However, all of them recovered by Day 7.

We first used a modified Irwin test^32,33^ to monitor neurobiological and physiological changes in Sprague-Dawley rats after they were subjected to repeated FUS-BBB opening. We have shown previously that a FUS exposure level of 0.8-MI could produce consistent BBB opening with no observable or occasional minimal erythrocyte extravasation, while an acoustic pressure of 1.4-MI poses higher risk for large-scale erythrocyte extravasations^16^. Therefore we examined the behavior of SD rats subjected to repeated FUS-BBB opening at exposure levels of 0.47-MI (below threshold), 0.8-MI (threshold), and 1.4-MI (excessive exposure). The treatments were given once each on days 1/3, with a 48 hour interval. No animal mortality or moribundity occurred during the study period. Hypoactivity, ataxia and tremors were observed on 2 rats out of the 10 subjected to high FUS exposure level (1.4-MI) (Fig. 2A, Supplementary Table S1). These changes were transient after FUS exposure and the rats fully recovered during the remaining observation period. Increased response to touch, click and tail pinch were observed on 1 rat at day 3 after 2^nd^ exposure of FUS-BBB opening in the 1.4-MI exposure group. Another animal in the 1.4-MI exposure group showed asleep posture and reduced mobility on day 3 (Fig. 2B, Supplementary Table S2). However, all these effects disappeared by day 7, and all the animals fully recovered. There were no statistically significant differences between the treatment and control groups in the categorical endpoints of CNS assessment (Supplementary Table S3). Nor were there differences in the distance of hind limb splay, indicating no neuromuscular dysfunction.

### Frequent and repetitive FUS exposure does not cause significant change in physical parameters

**Figure 3:**
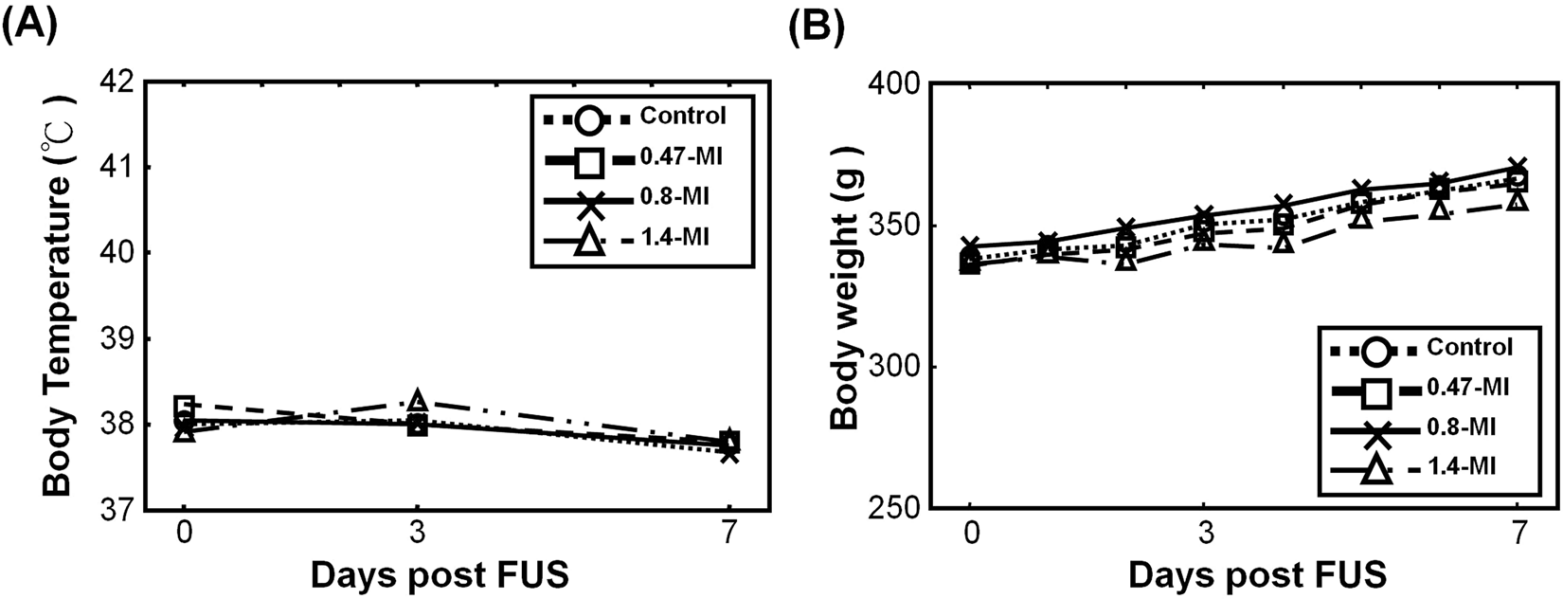
Records of mean body weight, mean body temperature, and plasma fibrinogen level. (**A**) Comparison of body temperature measured on Days −1/−2, Day 3, and Day 7 between groups (n = 10). A slight increase of rectal temperature was noted in the 1.4-MI exposure group on Day 3 after the 2^nd^ FUS application, but it did not reach statistical significance. By Day 7 all had returned to normal. (**B**) Comparison of body weight gain measured daily during observation period (n = 10). A trend toward reduced body weight gain was observed in the 1.4-MI exposure group, but it did not reach statistical significance.

We monitored other physiological parameters of SD rats subjected to FUS-BBB opening such as body weight, food consumption, body temperature, etc. There were no significant differences in terms of food consumption, rearing, defecation, and urination between the treatment and control groups during the observation period (Supplementary Table S4). A slight increase of rectal temperature of 38.3 °C was noted in the 1.4-MI exposure group on day 3 after second application of FUS, compared to 38.00 °C in the control group. However it did not reach statistical significance (Fig. 3A). Reduced body weight gain, down to 83.3% of the control group, was noted in 1.4-MI exposure group rats, but it did not reach statistical significance either (Fig. 3B).

### Frequent and repetitive high FUS exposure causes brain tissue damage without affecting other organs

**Figure 4:**
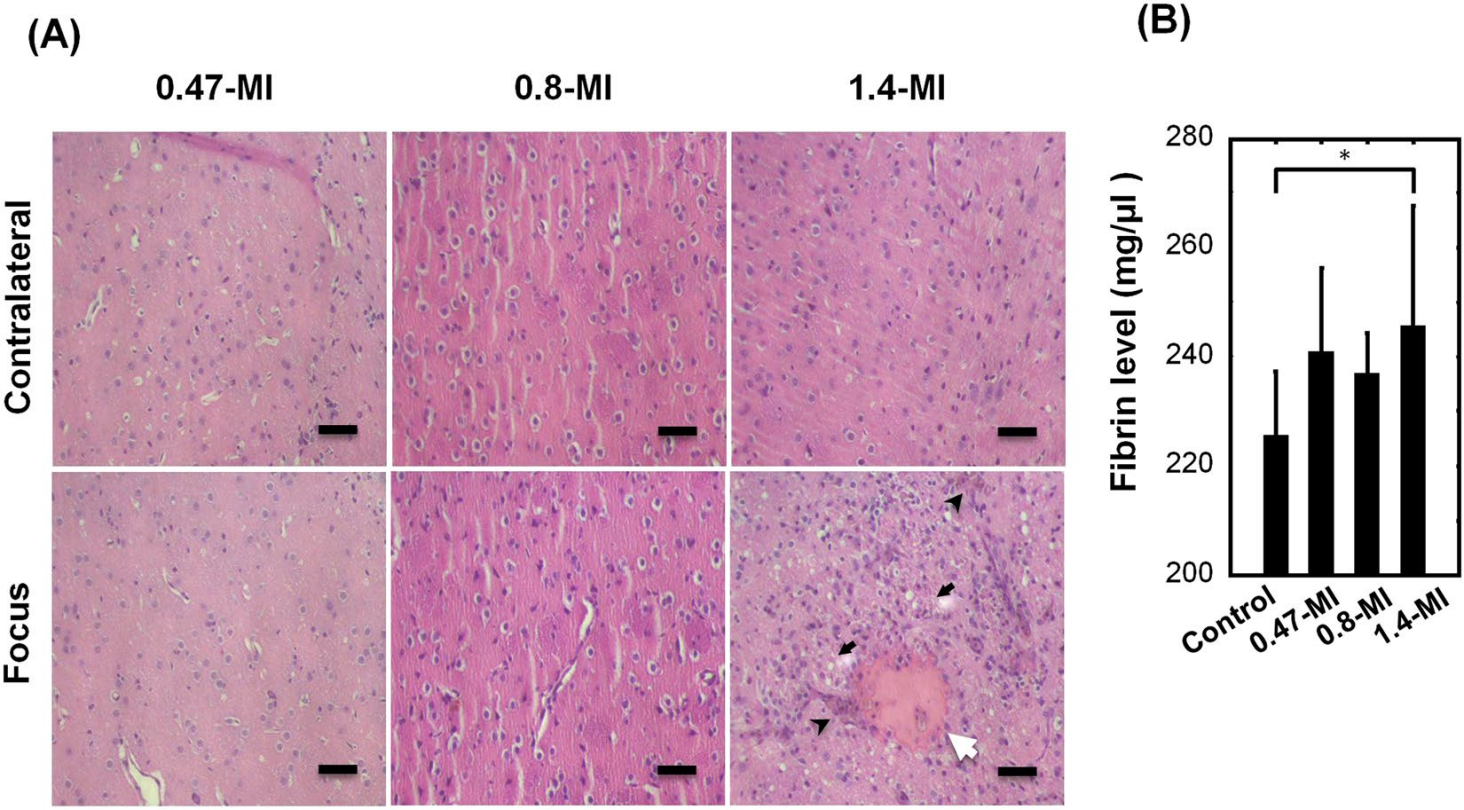
Representative hematoxylin-eosin-stained sections of FUS focus area and records of plasma fibrinogen level. (**A**) The samples were collected after 2 FUS-BBB openings on Days 1 and 3 and the animals were sacrificed on Day 8. Significant tissue damage was noted in the treated side in the 1.4-MI exposure group. n = 10. Scale bar: 50 µm, White arrow: fibrin deposition/hematoma, Black arrowhead: hemosiderin deposition, Black arrow: spotty necrosis. (**B**) Comparison of plasma fibrinogen concentration between 4 groups (mg/dL) collected on Day 8. Compared to the untreated group (Control), rats that received 2 1.4-MI exposures had significantly higher plasma fibrinogen levels compared to other groups; n = 10, *p < 0.05.

Histological examination of the brains of SD rats subjected to FUS-BBB opening revealed that 2 of the 10 rats receiving FUS treatment at 0.8-MI had minimal hemosiderin-laden macrophage, but no significant intracerebral hemorrhage was noted. The results indicated that repeated FUS-BBB opening at such acoustic pressure levels could induce mild erythrocyte leakage. On the other hand, 8 of the 10 rats subjected to 1.4-MI exposure had significant intracerebral hemorrhage (Fig. 4A, Table 3). Three of those 8 rats had tissue necrosis. The plasma fibrinogen level, an acute-phase reactant which is elevated in tissue damage or trauma, was significantly higher in the 1.4-MI exposure group than in the other groups (Fig. 4B). This result corresponds with the histology finding that FUS with high acoustic pressure is associated with intracerebral hemorrhage and brain damage. Examinations of other serum haematology, biochemistry, and other organs did not reveal any significant change (Supplementary Tables S5 and S6).

**Table 3:** Summary of abnormal observations in brain after FUS exposure.

Group		1 (0-MI)	2 (0.47-MI)	3 (0.8-MI)	4 (1.4-MI)
FUS strength		None	Light	Moderate	Heavy
Macrophage infiltration	Minimal Mild	0/10 0/10	0/10 0/10	2/10 0/10	5/10 3/10
Parenchymal loss	Minimal Mild	0/10 0/10	0/10 0/10	2/10 0/10	5/10 3/10
Necrosis	Minimal Mild	0/10 0/10	0/10 0/10	0/10 0/10	3/10 0/10

### Frequent and repetitive high FUS exposure with excessive microbubble dose induces cellular apoptosis

**Figure 5:**
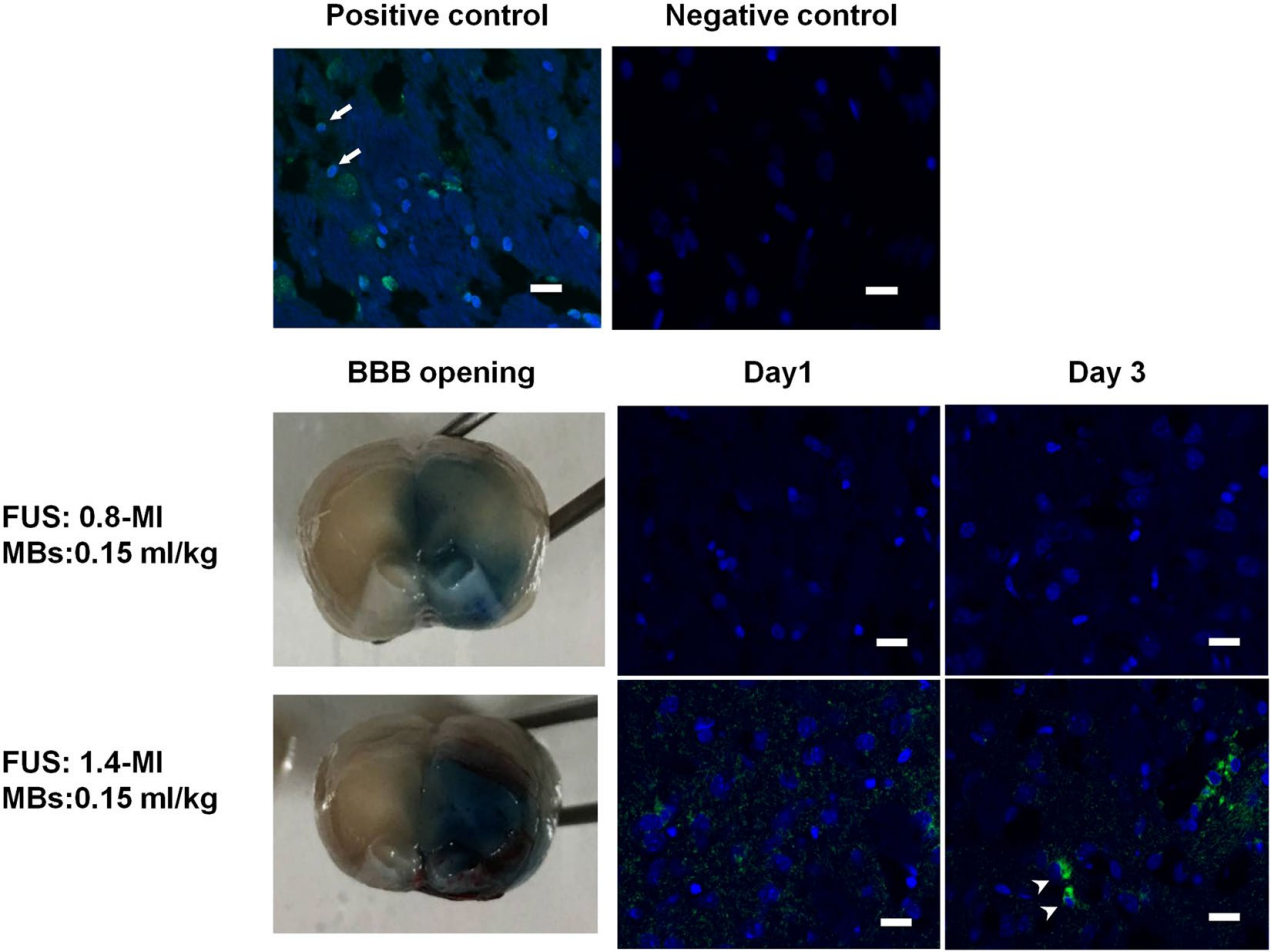
Representative TUNEL assay of the FUS focus area in the standard microbubble dose group. Rats were treated with one or two instances of either 0.8- or 1.4-MI FUS-BBB opening. All were given 0.15 ml/kg of microbubbles prior to FUS application. Positive TUNEL assay is represented by co-localization of blue and green fluorescence in the nucleus, while green fluorescence in the cytoplasm is viewed as artifact. Cellular apoptosis was not detected in either the 0.8- or the 1.4-MI exposure groups. n = 6. Scale bar: 20 µm, Blue: DAPI. Green: TUNEL assay. Arrow: positive TUNEL assay. Arrowhead: artifact.

**Figure 6:**
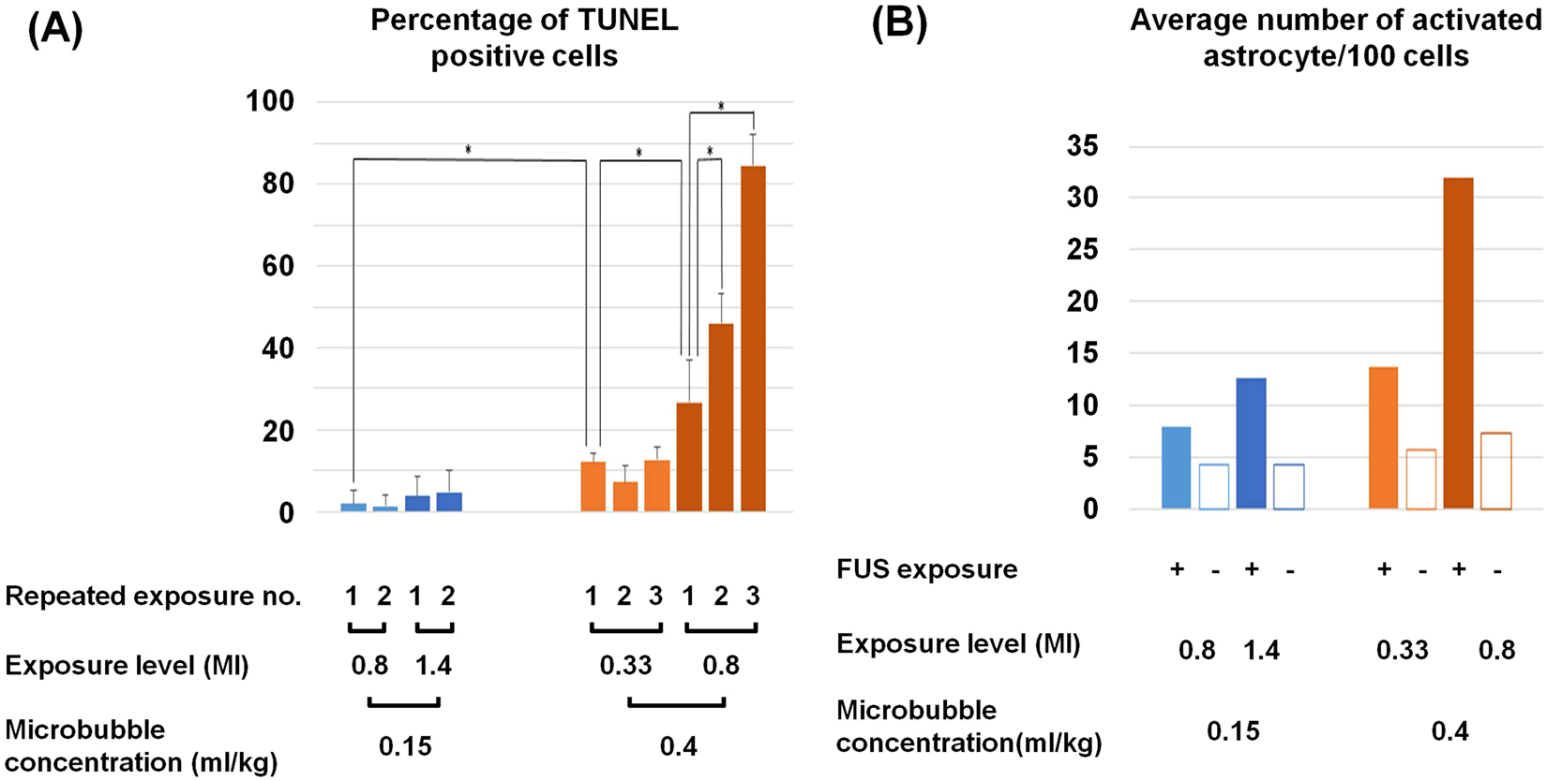
Quantification of TUNEL assay and GFAP expression. (**A**) Percentage of TUNEL positive cells. FUS-BBB opening protocols using excessive MB concentration results in statistically significant increase of cellular apoptosis. Also the combination of excessive MB with excessive FUS exposure results in cumulative ell damage. (**B**) Average number of activated astrocyte per 100 cells. In both normal MB and excessive MB group FUS-BBB opening induced astrocyte activation. n = 6. *p < 0.05.

In standard microbubble concentration administration (0.15 mL/kg; equivalents to 4 × 10^7^ MBs/kg) under frequent and repetitive FUS exposure, we found that application of FUS at 0.8-MI exposure produces BBB opening, as illustrated by the staining of Evans blue (EB) dye, while 1.4-MI exposure produces severe intraparenchymal hemorrhage accompanying this BBB-opening. However, neither 0.8- nor 1.4-MI FUS treatment (Groups 5–8) induces significant apoptosis (Figs 5, 6A). Recent studies have reported cellular apoptosis induced by FUS-BBB opening with low exposure levels (yet with the administration of a higher microbubble dose^22,23^) without inducing erythrocyte leakage. We therefore attempted to compare the effect of microbubble dosage on brain tissue when using FUS-BBB opening to achieve similar degrees of BBB opening effect.

**Figure 7:**
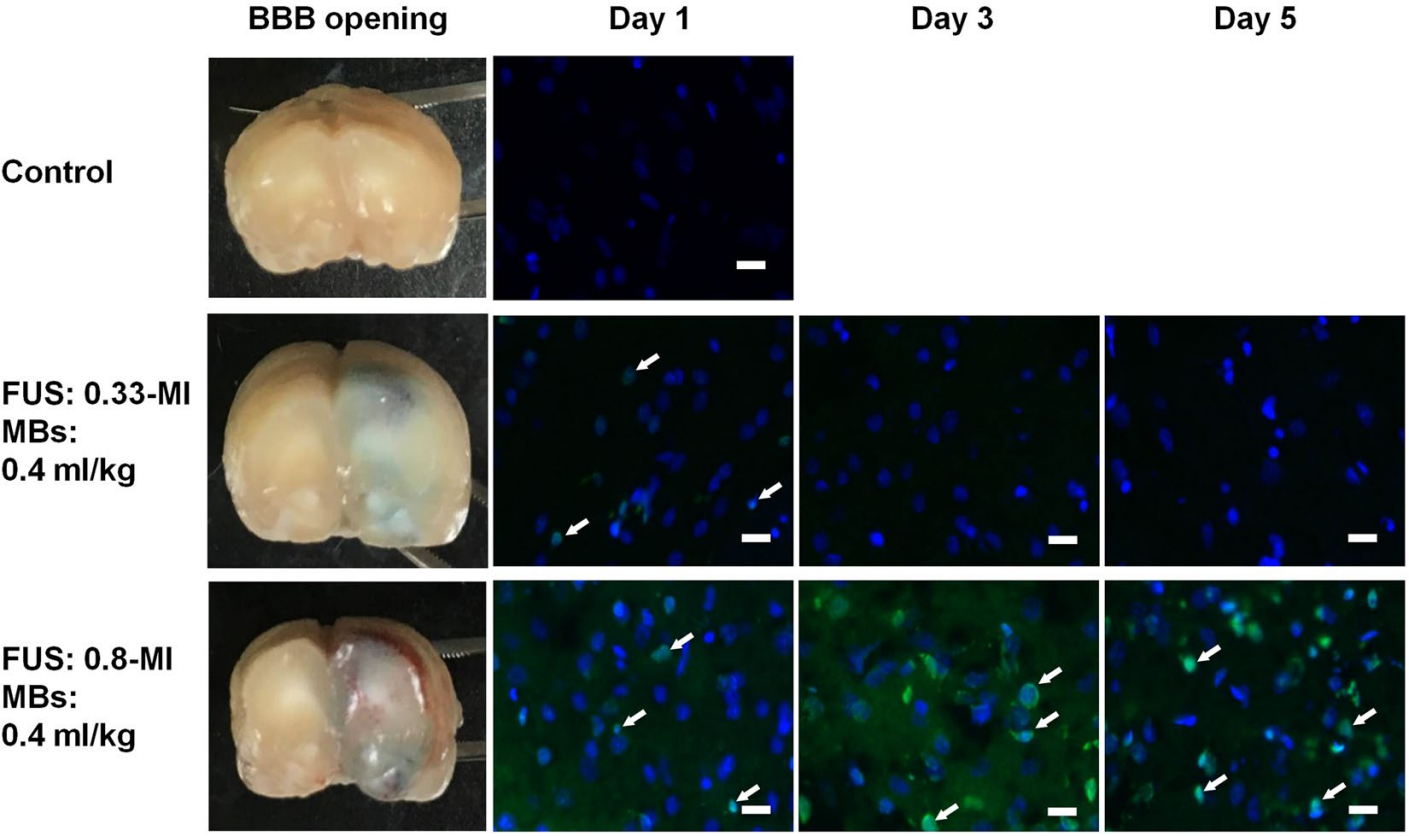
Representative TUNEL assay of FUS focus area in excessive microbubble dose group. Rats were treated with one to three episodes of either 0.33-MI or 0.8-MI FUS-BBB opening. All were given 0.4 ml/kg of microbubbles prior to FUS application. In the 0.33-MI group, a weak positive TUNEL assay could be detected after the first sonication, but was not detected after the second and third episodes. In the 0.8-MI exposure group, a strong positive TUNEL assay could be seen after first sonication, and progressively increased after subsequent episodes. n = 6. Scale bar: 20 µm, Blue: DAPI. Green: TUNEL assay. Arrow: positive TUNEL assay.

To evaluate the effect of excessive microbubble dosage on BBB opening, we increased the MB dosage to 0.4 mL/kg (equivalent to 2.0 × 10^8^ MBs/kg; Groups 9–14), used different FUS exposure levels to induce BBB opening, and examined the tissue effect. We found that in the 0.4 mL/kg group, 0.33-MI of FUS exposure was sufficient to induce BBB opening without significant erythrocyte extravasation, while 0.8-MI of FUS exposure induced gross intracerebral hemorrhage (Fig. 7). In terms of the BBB opening effect, 0.15 mL/kg MB/0.8-MI FUS induced results similar to 0.4 mL/kg MB/0.33-MI FUS, while 0.15 mL/kg MB/1.4-MI FUS was similar to 0.4 mL/kg MB/0.8-MI FUS.

Next we compared the induction of apoptosis between these two protocols. We found that by increasing microbubble concentration, FUS-BBB opening induced apoptotic change despite the reduced acoustic pressure. Specifically, the FUS protocol with acoustic pressure at 0.33-MI exposure in combination with 0.4 mL/kg microbubbles produced a BBB opening similar to that of FUS at 0.8-MI exposure and a microbubble concentration of 0.15 mL/kg (Groups 9–10 vs. Groups 5–6). And 0.8-MI FUS exposure in combination with 0.4 mL/kg produced intraparenchymal hemorrhage accompanying BBB opening, the same as 1.4-MI exposure with 0.15 mL/kg microbubbles (Groups 12–13 vs. Group 7–8). Specifically, the 0.15 mL/kg MB administration group did not induce apoptosis, while the 0.4 mL/kg MB administration group induced apoptosis in both acoustic pressure settings (Figs 6A, 7). To evaluate whether the effect was cumulative after frequently repeated FUS application, we performed FUS-BBB opening on Days 1/3/5 using the 0.4 mL/kg microbubble protocols (Groups 11 and 14). We found that, while cellular apoptosis appeared in the 0.33-MI/0.4 mL/kg group (Group 11) after performing the first FUS exposure, it was not apparent after the 2^nd^ (Day 3) and 3^rd^ (Day 5) exposure. On the other hand, TUNEL assay staining was clearly visible in the 0.8-MI/0.4 ml/kg group (Group 14) samples, and progressively increased after repeated FUS application (Fig. 7). Also double staining showed colocalization of Annexin V and Synaptophysin, indicating neuronal apoptosis (Supplementary Fig. S2). The results indicate that the effect was cumulative at such FUS exposure settings given 0.8-MI exposure with high concentration MB administration. In addition, it also indicates that the cellular apoptosis induced by FUS-BBB opening is not solely proportional to acoustic exposure level, but might be related to other factors such as microbubble dosage.

### FUS - BBB opening induces astroglial cell activation

**Figure 8:**
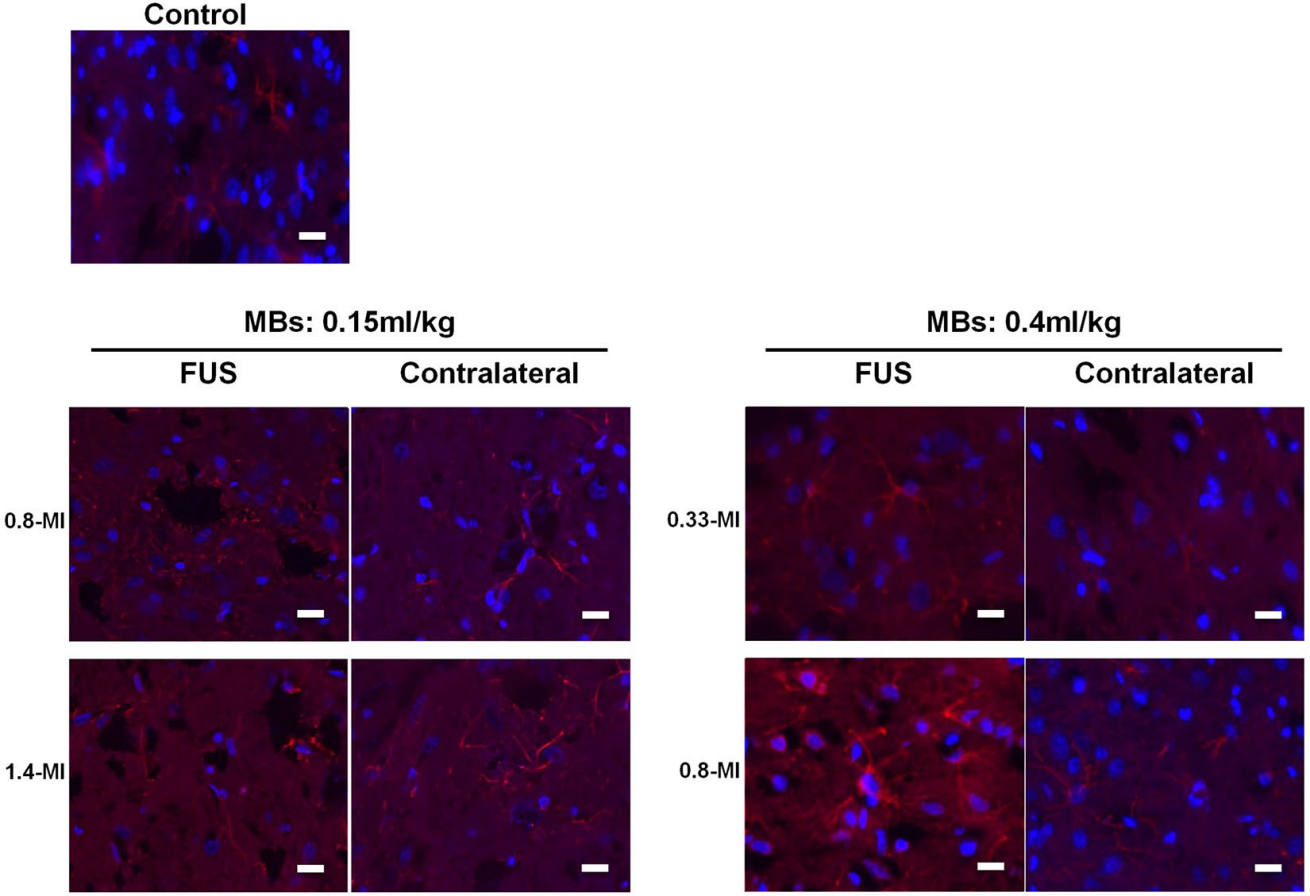
Representative GFAP staining of FUS focus area. GFAP staining of FUS focus of “moderate” (0.15 ml/kg microbubbles-0.8-MI and 0.4 ml/kg microbubbles-0.33-MI) and “heavy” (0.15 ml/kg microbubbles-1.4-MI and 0.4 ml/kg microbubbles-0.8-MI) group after 2 exposures. While only weak GFAP staining could be detected in the control group and on the untreated side (contralateral), strong GFAP staining could be seen on the treated focus area in all groups. The results indicated that both FUS-BBB opening protocols with either “moderate” or “heavy” exposure induced astroglial activation in the focus area. n = 6. Blue: DAPI, Red: GFAP. Scale bar = 20 µm.

In addition to the TUNEL assay, we also examined astroglial activation of the rat brain at the focus after two exposures. We stained the rat brains subjected to both 0.15 mL/kg or 0.4 mL/kg MBs protocols with either moderate or heavy FUS-BBB opening for GFAP (Group 6, 8, 10 or 13). As shown in Fig. 8, both the 0.15 mL/kg and 0.4 mL/kg protocols induced astroglial activation on the treatment side compared to the untreated side (Fig. 6B). These results indicate that FUS-BBB opening induced astroglial activation and glial scar formation.

## Discussion

Glioblastoma is the most malignant and most common primary malignant brain tumor, and blood-brain barrier is a severe impediment to effectively deliver therapeutic agent for glioblastoma treatment. Current standard treatment comprises of total excision of the tumor, followed by concurrent chemo-radiotherapy (CCRT) and 6 months of oral chemotherapy^25,34^. The prognosis is poor despite aggressive treatment, with median survival 14.1 months, one year survival rate 37.2%, and 5 year survival rate 5.1%^3,35^. Of these treatment modalities, surgical resection is most effective in increasing patient survival^36,37^. The addition of temozolomide CCRT and chemotherapy increases median survival by roughly 2 months. We designed the experiments based on the assumption that FUS-BBB opening is an adjunct to chemotherapy. Previous works have established the effectiveness of FUS treatment in enhancing chemotherapeutic delivery, improving tumor control, and increasing survival of tumor bearing mice^17,18^. The effect was explained by the ability of FUS-BBB opening to enhance drug delivery to the peripheral normal brain tissue while improving drug retain in the tumor core without posting other biological hazard such as intracerebral hemorrhage^31,38^. In addition to improve drug delivering efficiency to brain tumor, FUS-BBB opening may offer physicians the chance to design protocols with less frequent injections or reduced dose to reduce systemic toxicity while enhance tumor control.

Our previous results have shown consistently that FUS-BBB opening in brain-tumor-bearing mice was less effective in increasing drug delivery to tumor tissue compared to tumor periphery, mainly due to the “leaky” vasculature compared to normal one. However, FUS-BBB opening significantly increased drug delivery to the normal brain areas peripheral to the main tumor^31^, which is the major area of tumor recurrence, contains normal BBB, and shields tumor cells from drug treatment, and is suggested to be the major location to be treated by FUS. This is one of the key rationale that we performed FUS-BBB opening on normal mice to simulate the effect on the periphery of brain tumor. Our results suggested that performing FUS-BBB opening every other day should be also safe at tumor peripheral, given careful monitoring and adjustment of FUS parameters.

In our study, modified Irwin’s test was used to evaluate the CNS toxicity of repeated FUS-BBB opening. We found that repeated FUS-BBB opening procedure every other day using threshold or below-threshold FUS intensity produced no observable behavior change, while rats in the ‘excessive FUS exposure’ group showed minor and transient change in both physical condition and behavior. There was no morbidity or moribundity, which affirms protocol safety. Physically, a trend toward slightly elevated body temperature was observed on day 3, along with reduced body weight gain throughout study in the “excessive FUS exposure’ group, but neither phenomenon reached statistical significance. Behaviorally, the ‘excessive FUS exposure’ group showed mild transient hypoactivity, ataxia, and tremors that disappear on Day 8. These results may be due to brain damage, tissue necrosis or intraparenchymal hemorrhage induced by high intensity of FUS. The lack of significant long-term change at such high intensity FUS may be due to behavior adaptation or compensation. These results suggest that frequent repeated FUS-BBB opening at the adequate FUS exposure level did not cause significant brain function changes in rats. Due to resource constraints provided by the certified testing environment, we were unable to perform further animal behavior tests to evaluate specific aspects of higher cortical function such as memory, spatial ability, attention, or emotional status. Further animal behavior tests such as water maze or forced swim test are required to evaluate effects of FUS-MB BBB opening on specific aspects of higher cortical function such as learning, memory, or emotion.

Earlier papers studying the safety of FUS-BBB opening focused on animal behavior and histological findings^39,40^. The protocol for FUS administration varies between testing animal species and exposure conditions, but most agree that, with careful control, an optimal FUS intensity, frequency, and microbubble concentration can be found to produce adequate BBB opening without causing identifiable histological damages or produces only minor erythrocyte leakage which is cleared within a few days. These studies also reported no increase of cellular damage or apoptosis with microbubble dosages within the range recommended for ultrasound imaging contrast^39–41^. In addition, these studies also reported no identifiable animal behavioral changes within the adequate FUS exposure group, and only minor behavior changes given ‘heavy’ FUS treatment^29,40^. However two recent studies have shown that even moderate FUS exposure, which does not increase risk of intra-parenchymal hemorrhage, is still a risk for cellular apoptosis and sterile inflammation^22,23^. Kovacs *et al*. reported that application of FUS-BBB opening with an MB dosage roughly equivalent to 2 × 10^8^ MBs/kg produces sterile inflammation, with increases of both pro-inflammatory and anti-inflammatory cytokines^22^. McMahon *et al*. showed that the acute inflammatory response and cellular apoptosis is dependent on microbubble dose, with MB dose roughly equivalent to 1.2 × 10^9^ MBs/kg associated with strong NF-kB pathway activation, inflammatory response, and apoptosis^23^. An early report by Vykhodtseva *et al*. indicated that apoptosis can be induced with a much lower MB dosage given sufficient FUS exposure to cause tissue heating, with the pro-apoptotic effect appeared to be amplified by intracerebral hemorrhage^42^. These studies have shown that either excessive microbubbles or excessive FUS exposure induces apoptosis. However these studies were performed with either fixed MB concentration or fixed sonic pressure. As the parameter of FUS-MB BBB opening changed, so did its biological effect, namely the degree of BBB opening. We tried to elucidate whether adjust sonic pressure or microbubble concentration to achieve similar degree of BBB opening affect its ability to induce apoptosis. We set two biological “endpoints”, BBB opening without intracerebral hemorrhage and BBB opening with intracerebral hemorrhage. The FUS parameters were determined and optimized based on our previous experiments^43,44^. We found that the BBB opening effect was observed in both the ‘standard MB dosage’ group (equivalent to 4 × 10^7^ MBs/kg) and the ‘excessive MB dosage’ group (equivalent to 2.0 × 10^8^ MBs/kg) after threshold ‘adequate’ FUS intensity exposure. Significant BBB opening and intensive intracerebral hemorrhages in the focus area were also observed in both groups after above-threshold ‘excessive’ FUS exposure. Both produced astrocyte activation and reactive gliosis, which were also noted in previous reports^22,45^. However, we observed that in both “Moderate” and ‘Heavy” FUS exposure groups, ‘excessive MB dosage’ groups have significantly more apoptotic cells than in ‘standard MB dosage’ group. Our results indicate that even with similar degree of BBB opening, protocols involving higher MB concentration are more likely to induce apoptosis.

In our experimental design, we include groups of heavy FUS exposure which induces intracerebral hemorrhage to simulate inadvertent delivery of excessive acoustic pressure to a focal point. As expected, excessive FUS exposure resulted in increased apoptosis and increased tissue damage. Rats that received excessive FUS exposure had significantly more tissue necrosis and hemosiderin laden macrophage infiltration in the brain FUS focus area, and had significantly higher serum fibrinogen. Furthermore, in ‘excessive MB dosage’ with ‘heavy FUS exposure’ group the pro-apoptotic effect appeared to be cumulative. As the number of sonication increased, so did the percentage of apoptotic cells. The effect was not observed in either ‘normal MB dosage’ groups or in ‘moderate FUS exposure’ group of ‘excessive MB dosage’ group. We surmise the effect might be related to the inflammatory response induced by FUS-BBB opening.

Previous study has shown that FUS-BBB opening induced an early increase of pro-inflammatory cytokines and delayed increase of anti-inflammatory cytokines and neurotrophic factors^22^. The anti-inflammatory cytokines may contribute to neuroprotection and limit tissue damage against further sonication episodes. This biological response has been confirmed due to the mechanical stress relating to acoustic cavitation produced during ultrasound-microbubble interaction. The scale of BBB opening highly correlates with overall cavitation activity, whereas the inertial cavitation, occurred during a more violate microbubble disrupt, and is more likely responsible to cause cellular damage or induce biological response such as inflammation in the brain^46,47^. It is also known that the threshold of exposure level to trigger significant inertial cavitation sharply decreases as the microbubble concentration increases^48^. Therefore, it is conceivable that high MB concentration group might have produced excessive inertial cavitation effect and contributed to biological response other than BBB opening, including the induction of higher percentage of apoptotic cells. Furthermore, combining excessive FUS exposure with excessive MB might have resulted in increased tissue damage, increased expression of damage associated molecular pattern and increased inflammatory response which surpassed the protective effect of the anti-inflammatory cytokines, thus the cumulative effect.

A passive cavitation detection (PCD) device could help to delineate the strength of cavitation activity to correlate these phenomena. However we were unable to perform PCD monitoring because part of our experiments were carried out in a certified third-party facility for objectivity and fairness. Due to the operator-dependent nature of PCD and the difficulty of establishing PCD hardware in a standardized lab, we were forced to forgo the monitoring and determine FUS parameters based on our previous experiments^43,44^. In a clinical setting such monitoring procedure should be considered a necessity.

Gliosis is a reactive change of glial cells in the central nervous system in response to tissue damage. It is characterized by an intense activation of CNS microglia. We attempted to conduct ED1/IBA1 staining to confirm the presence of macrophage/microglia activation, but no significant microglia activation been observed (This examination is not presented in this manuscript). Neither staining showed significant increase at the focus of the FUS site. There was an increase in the number of activated astrocyte at the site of focus, but the cell density and intensity were much lower than expected for gliosis. In our previous study, we have shown that application of FUS may induce tiny areas of gliosis in tumor bearing mice 75 days after FUS application^17^. Though the FUS application parameters were similar to our low microbubble concentration group, the gliosis was located immediately adjacent to the tumor^17^. The microenvironment change induced by tumor and the chronicity of experiment design might be the cause of the difference between experiments. Further study is required to evaluate the effect of microbubble concentration on the ability of FUS to induce gliosis or glial scaring.

In our study, we show that FUS-BBB opening protocols involving MB doses exceeding 1.2 × 10^8^ MBs/kg induced apoptosis, while those using MB doses of up to 4.5 × 10^7^ MBs/kg may be safe. These results are consistent with the results of prior studies^22,23^. It should be noted that all these studies, including ours, used different types of microbubbles to achieve BBB opening. They differ in terms of shell composition (Optison: human serum albumin, Definity: lipid, Sonovue: lipid), gas content (Optison: perflutren, Definity: octafluopropane, Sonovue: sulphur hexafluoride), microbubble concentration (Optison: 5~8 × 10^8^/ml, Definitiy: 1.2 × 10^10^/ml, Sonovue: 1 ~ 5 × 10^7^/ml) and mean diameter (Optison: 3.0–4.5 μm; Definity: 1.1–3.3 μm, SonoVue: 1.5–2.5 μm). These factors may affect their interaction with FUS, their ability to induce inflammation and induce apoptosis. Other factors such as the variation of capillary blood flow and capillary diameter post variation in regional neuronal activity, blood CO_2_ concentration, endothelial functions, etc.^49^, and as consequence potentially affect local microbubble concentration.

Anesthesia protocol during FUS-BBB opening is also a potential factor that needs to be considered. In our experiment, we used oxygen as the carrier gas, and used 2–2.5% isoflurane. The anesthesia depth was titrated based on the respiration rate. When taking anesthetic protocol into consideration, both the selection of inhalational anesthetics and the carrying gas affect intravascular microbubbles volume due to the varying microbubble dissolution dynamics and capillary contraction/dilation behavior^50,51^. It not only posts effect on FUS-BBB opening efficiency, but also significantly affects the microbubble concentration and resulting tissue damage^52^. In addition, previously it has been reported that different volatile anesthetics modulates tight junction protein expression and affect BBB permeability, further compounds the problem^53^. These factors when compounding together make comparison between studies even more difficult with the BBB opening effect to be quantitated, and should be further investigated.

While comparing our results to other studies, we draw common observation that FUS-BBB opening with MB dosage exceeding 10^8^ MBs/kg may post hemorrhagic and pro-inflammatory effect after repetitively FUS exposure, whereas below 10^7^ MBs/kg the brain after repetitively FUS exposure is intact. Although characterizing a definite ‘safe’ cut-off value of microbubble concentration is challenging due to the highly dynamic nature of microbubble circulation, it may give us an useful suggestion to roughly gauge the microbubble usage to avoid potential tissue damage relating to inflammation or cellular apoptosis.

## Conclusion

In conclusion, we have shown that frequent application of FUS-BBB opening with a well-controlled exposure level is a safe procedure. Repeated application of FUS-BBB opening every 2 days with moderate acoustic pressure induced adequate BBB opening without causing tissue damage or behavioral change, while FUS with acoustic pressure significantly higher than required caused minor and transient behavior change. FUS-BBB opening with microbubble dosages exceeding 1.2 × 10^8^ MBs/kg may induce cellular apoptosis, while microbubble dosages up to 4.5 × 10^7^ MBs/kg may be safe provided adequate FUS exposure. Fine-tuning the BBB-FUS protocol is required to maximize its BBB opening effect and minimize potential cellular damage. This study suggests that frequently repeated FUS-BBB opening could be safe given carefully calibrated FUS exposure levels.

### Ethics approval and consent to participate

All animal procedures were approved by Chang Gung University IACUC board under IACUC No. CGU14-136.

## Electronic supplementary material

Supplementary Information
