# Supplementary material for: Safety evaluation of frequent application of microbubble-enhanced focused ultrasound blood-brain-barrier opening
Source: Sci Rep. 2018 Dec 7;8:17720. doi: 10.1038/s41598-018-35677-w (PMC6286368; doi:10.1038/s41598-018-35677-w)
Supplement: Supplementary file 1 — Supplementary Information [file 41598_2018_35677_MOESM1_ESM.docx]

# Safety evaluation of frequent application of microbubble-enhanced focused ultrasound blood-brain-barrier opening

Hong-Chieh Tsai^1, 2^* <[newcomer9999@gmail.com](mailto:newcomer9999@gmail.com)>, Chih-Hung Tsai^3^* <[chiuhung_tsai@hotmail.com](mailto:chiuhung_tsai@hotmail.com)> , Wen-Shiang Chen^4^ <[wenshiang@gmail.com](mailto:wenshiang@gmail.com)>, Claude Inserra^5,6^ <[claude.inserra@inserm.fr](mailto:claude.inserra@inserm.fr)>, Kuo-Chen Wei^1^**<[kuochenwei@cgmh.org.tw](mailto:kuochenwei@cgmh.org.tw)>, and Hao-Li Liu^1, 3^**<[haoliliu@mail.cgu.edu.tw](mailto:haoliliu@mail.cgu.edu.tw)>

^1^Department of Neurosurgery, Chang Gung Memorial Hospital at Linkou, Taoyuan, 333 Taiwan.

^2^Graduate Institute of Clinical Medical Sciences of Chang Gung University, Taoyuan, 333 Taiwan.

^3^Department of Electrical Engineering, Chang-Gung University, Taoyuan, 333 Taiwan

^4^Department of Physical Medicine & Rehabilitation, National Taiwan University Hospital, Taipei 100 Taiwan

^5^Inserm, U1032, Lyon, F-69003, France; Université de Lyon, Lyon, F-69003, France

^6^Institut Lumière Matière, UMR 5306, Université Lyon 1-CNRS, Université de Lyon, 69622 Villeurbanne cedex, France

*H-C Tsai and C-H Tsai contributed equally to this work.

**Address correspondence to:

Hao-Li Liu, Ph.D.

Department of Electrical Engineering, Chang Gung University

259 Wen-Hwa 1st Road, Taoyuan, 333, Taiwan.

Department of Neurosurgery, Chang Gung Memorial Hospital at Linkou, Taoyuan, 333 Taiwan.

Tel: +886-3-2118800 ext 5715. Fax: +886-3-2118026.

E-mail: [haoliliu@mail.cgu.edu.tw](mailto:haoliliu@mail.cgu.edu.tw)

Kuo-Chen Wei, M.D.

Department of Neurosurgery, Chang Gung Memorial Hospital, Taoyuan at Linkou, Taiwan.

5 Fu-Hsin 1^st^ , Taoyuan, 333, Taiwan

Tel: +886-3-3281200 ext 2412.

E-mail: [kuochenwei@cgmh.org.tw](mailto:kuochenwei@cgmh.org.tw)

# Supplementary Information

## Table S1. Summary of Mortality and Clinical Observations.

| **Items** | **Exposure level (MI)** | **Incidence (Occurrence/ total)** | | | | | | |
| --- | --- | --- | --- | --- | --- | --- | --- | --- |
|  |  | Day 1 | Day 2 | Day 3 | Day 4 | Day 5 | Day 6 | Day 7 |
| Mortality | 0 | 0/10 | 0/10 | 0/10 | 0/10 | 0/10 | 0/10 | 0/10 |
|  | 0.47 | 0/10 | 0/10 | 0/10 | 0/10 | 0/10 | 0/10 | 0/10 |
|  | 0.8 | 0/10 | 0/10 | 0/10 | 0/10 | 0/10 | 0/10 | 0/10 |
|  | 1.4 | 0/10 | 0/10 | 0/10 | 0/10 | 0/10 | 0/10 | 0/10 |
| Hypoactivity | 0 | 0/10 | 0/10 | 0/10 | 0/10 | 0/10 | 0/10 | 0/10 |
|  | 0.47 | 0/10 | 0/10 | 0/10 | 0/10 | 0/10 | 0/10 | 0/10 |
|  | 0.8 | 0/10 | 0/10 | 0/10 | 0/10 | 0/10 | 0/10 | 0/10 |
|  | 1.4 | 2/10 | 1/10 | 1/10 | 0/10 | 0/10 | 0/10 | 0/10 |
| Ataxia | 0 | 0/10 | 0/10 | 0/10 | 0/10 | 0/10 | 0/10 | 0/10 |
|  | 0.47 | 0/10 | 0/10 | 0/10 | 0/10 | 0/10 | 0/10 | 0/10 |
|  | 0.8 | 0/10 | 0/10 | 0/10 | 0/10 | 0/10 | 0/10 | 0/10 |
|  | 1.4 | 1/10 | 0/10 | 0/10 | 0/10 | 0/10 | 0/10 | 0/10 |
| Tremors | 0 | 0/10 | 0/10 | 0/10 | 0/10 | 0/10 | 0/10 | 0/10 |
|  | 0.47 | 0/10 | 0/10 | 0/10 | 0/10 | 0/10 | 0/10 | 0/10 |
|  | 0.8 | 0/10 | 0/10 | 0/10 | 0/10 | 0/10 | 0/10 | 0/10 |
|  | 1.4 | 1/10 | 0/10 | 0/10 | 0/10 | 0/10 | 0/10 | 0/10 |
| Swelling-area of forehead | 0 | 0/10 | 0/10 | 0/10 | 0/10 | 0/10 | 0/10 | 0/10 |
|  | 0.47 | 0/10 | 0/10 | 0/10 | 0/10 | 0/10 | 0/10 | 0/10 |
|  | 0.8 | 0/10 | 0/10 | 0/10 | 0/10 | 0/10 | 0/10 | 0/10 |
|  | 1.4 | 1/10 | 0/10 | 0/10 | 0/10 | 0/10 | 0/10 | 0/10 |
| Urine stain | 0 | 0/10 | 0/10 | 0/10 | 0/10 | 0/10 | 0/10 | 0/10 |
|  | 0.47 | 0/10 | 0/10 | 0/10 | 0/10 | 0/10 | 0/10 | 0/10 |
|  | 0.8 | 0/10 | 0/10 | 0/10 | 0/10 | 0/10 | 0/10 | 0/10 |
|  | 1.4 | 1/10 | 0/10 | 0/10 | 0/10 | 0/10 | 0/10 | 0/10 |
| Chromo-dacryorrhea | 0 | 0/10 | 0/10 | 0/10 | 0/10 | 1/10 | 0/10 | 0/10 |
|  | 0.47 | 0/10 | 0/10 | 0/10 | 0/10 | 0/10 | 0/10 | 0/10 |
|  | 0.8 | 0/10 | 0/10 | 0/10 | 0/10 | 0/10 | 0/10 | 0/10 |
|  | 1.4 | 0/10 | 1/10 | 0/10 | 0/10 | 0/10 | 0/10 | 0/10 |
| Wounds-area of forehead | 0 | 0/10 | 0/10 | 0/10 | 0/10 | 0/10 | 0/10 | 0/10 |
|  | 0.47 | 0/10 | 0/10 | 0/10 | 0/10 | 0/10 | 0/10 | 0/10 |
|  | 0.8 | 0/10 | 1/10 | 2/10 | 2/10 | 2/10 | 2/10 | 1/10 |
|  | 1.4 | 0/10 | 1/10 | 2/10 | 2/10 | 1/10 | 0/10 | 0/10 |
| Hair loss-forepaw | 0 | 0/10 | 0/10 | 0/10 | 0/10 | 0/10 | 0/10 | 0/10 |
|  | 0.47 | 0/10 | 0/10 | 0/10 | 0/10 | 0/10 | 0/10 | 0/10 |
|  | 0.8 | 0/10 | 0/10 | 0/10 | 0/10 | 0/10 | 0/10 | 0/10 |
|  | 1.4 | 0/10 | 1/10 | 1/10 | 1/10 | 1/10 | 1/10 | 1/10 |

## Table S2. Summary of Descriptive Endpoints during Observation Period.

| **Descriptive Endpoint** | **Exposure level (MI)** | **Incidence during observation period (n'/n')** | | |
| --- | --- | --- | --- | --- |
|  |  | Day -2/-1 prior to exposure | Day 3 | Day 7 |
|  |  |  |  |  |
|  |  |  |  |  |
| Posture: Sitting/standing | 0 | 10/10 | 10/10 | 10/10 |
|  | 0.47 | 10/10 | 10/10 | 10/10 |
|  | 0.8 | 10/10 | 9/10 | 10/10 |
|  | 1.4 | 9/10 | 8/10 | 10/10 |
|  |  |  |  |  |
| Alert | 0 | 0/10 | 0/10 | 0/10 |
|  | 0.47 | 0/10 | 0/10 | 0/10 |
|  | 0.8 | 0/10 | 1/10 | 0/10 |
|  | 1.4 | 1/10 | 1/10 | 0/10 |
|  |  |  |  |  |
| Asleep | 0 | 0/10 | 0/10 | 0/10 |
|  | 0.47 | 0/10 | 0/10 | 0/10 |
|  | 0.8 | 0/10 | 0/10 | 0/10 |
|  | 1.4 | 0/10 | 1/10 | 0/10 |
|  |  |  |  |  |
| Stereotype |  |  |  |  |
| Tremor | 0 | 0/10 | 0/10 | 0/10 |
|  | 0.47 | 0/10 | 0/10 | 0/10 |
|  | 0.8 | 0/10 | 0/10 | 0/10 |
|  | 1.4 | 0/10 | 0/10 | 0/10 |
|  |  |  |  |  |
| Bizarre behaviors |  |  |  |  |
| Tremor | 0 | 0/10 | 0/10 | 0/10 |
|  | 0.47 | 0/10 | 0/10 | 0/10 |
|  | 0.8 | 0/10 | 0/10 | 0/10 |
|  | 1.4 | 0/10 | 1/10 | 0/10 |

## Table S3. Summary of Categorical Endpoints during Observation Period.

| **Categorical Endpoint** | **Exposure level (MI)** | **Mean score (average of sum in categorical scored of each endpoint)** | | |
| --- | --- | --- | --- | --- |
|  |  | Day -2/-1 prior to exposure | Day 3 | Day 7 |
|  |  |  |  |  |
| Respiration | 0 | 1 | 1 | 1 |
|  | 0.47 | 1 | 1 | 1 |
|  | 0.8 | 1 | 1 | 1 |
|  | 1.4 | 1 | 1 | 1 |
| Clonic-involuntaryj | 0 | 1 | 1 | 1 |
|  | 0.47 | 1 | 1 | 1 |
|  | 0.8 | 1 | 1 | 1 |
|  | 1.4 | 1 | 1 | 1 |
| Tonic-involuntary | 0 | 1 | 1 | 1 |
|  | 0.47 | 1 | 1 | 1 |
|  | 0.8 | 1 | 1 | 1 |
|  | 1.4 | 1 | 1 | 1 |
| Vocali-zations | 0 | 1 | 1 | 1 |
|  | 0.47 | 1 | 1 | 1 |
|  | 0.8 | 1 | 1 | 1 |
|  | 1.4 | 1 | 1 | 1 |
| Piloerection | 0 | 1 | 1 | 1 |
|  | 0.47 | 1 | 1 | 1 |
|  | 0.8 | 1 | 1 | 1 |
|  | 1.4 | 1 | 1 | 1 |
| Palpebral closure | 0 | 1 | 1 | 1 |
|  | 0.47 | 1 | 1 | 1 |
|  | 0.8 | 1 | 1 | 1 |
|  | 1.4 | 1 | 1.1 | 1 |
| Ease to remove | 0 | 2 | 2 | 2 |
|  | 0.47 | 1.9 | 2 | 2 |
|  | 0.8 | 2 | 2 | 1.9 |
|  | 1.4 | 2 | 2 | 2 |
| Ease to handle | 0 | 2 | 1.9 | 2 |
|  | 0.47 | 2.2 | 1.9 | 2 |
|  | 0.8 | 1.9 | 2 | 1.9 |
|  | 1.4 | 2 | 2.1 | 2 |
| Chromodacryorrhea | 0 | 1 | 1 | 1 |
|  | 0.47 | 1 | 1 | 1 |
|  | 0.8 | 1 | 1 | 1 |
|  | 1.4 | 1 | 1 | 1 |
| Lacrimation | 0 | 1 | 1 | 1 |
|  | 0.47 | 1 | 1 | 1 |
|  | 0.8 | 1 | 1 | 1 |
|  | 1.4 | 1 | 1 | 1 |
| Salivation | 0 | 1 | 1 | 1 |
|  | 0.47 | 1 | 1 | 1 |
|  | 0.8 | 1 | 1 | 1 |
|  | 1.4 | 1 | 1 | 1 |
| Fur coat | 0 | 1 | 1 | 1 |
|  | 0.47 | 1 | 1 | 1 |
|  | 0.8 | 1 | 1 | 1 |
|  | 1.4 | 1 | 1 | 1 |
| Visual approach response | 0 | 1 | 1 | 1 |
|  | 0.47 | 1 | 1 | 1 |
|  | 0.8 | 1 | 1 | 1 |
|  | 1.4 | 1 | 1 | 1 |
| Touch response | 0 | 1 | 1 | 1 |
|  | 0.47 | 1 | 1 | 1 |
|  | 0.8 | 1 | 1 | 1 |
|  | 1.4 | 1 | 1.4 | 1 |
| Click response | 0 | 1.2 | 1 | 1 |
|  | 0.47 | 1.4 | 1 | 1 |
|  | 0.8 | 1 | 1 | 1 |
|  | 1.4 | 1.2 | 1.6 | 1 |
| Eyelid reflex | 0 | 1 | 1 | 1 |
|  | 0.47 | 1 | 1 | 1 |
|  | 0.8 | 1 | 1 | 1 |
|  | 1.4 | 1 | 1 | 1 |
| Pinna reflex | 0 | 1 | 1 | 1 |
|  | 0.47 | 1 | 1 | 1 |
|  | 0.8 | 1 | 1 | 1 |
|  | 1.4 | 1 | 1 | 1 |
| Tail pinch response | 0 | 1.2 | 1 | 1.4 |
|  | 0.47 | 1.8 | 1.4 | 1 |
|  | 0.8 | 1.2 | 1.2 | 1 |
|  | 1.4 | 1.4 | 2 | 1 |
| Pupil reflex | 0 | 1 | 1 | 1 |
|  | 0.47 | 1 | 1 | 1 |
|  | 0.8 | 1 | 1 | 1 |
|  | 1.4 | 1 | 1 | 1 |
| Proprioception | 0 | 1 | 1 | 1 |
|  | 0.47 | 1 | 1 | 1 |
|  | 0.8 | 1 | 1 | 1 |
|  | 1.4 | 1 | 1 | 1 |
| Air righting reflex | 0 | 1 | 1 | 1 |
|  | 0.47 | 1 | 1 | 1 |
|  | 0.8 | 1 | 1 | 1 |
|  | 1.4 | 1 | 1 | 1 |
| Abdominal tone | 0 | 2 | 2 | 2 |
|  | 0.47 | 2 | 2 | 2 |
|  | 0.8 | 2 | 2 | 2 |
|  | 1.4 | 2 | 2 | 2 |
| Limb tone | 0 | 2 | 2 | 2 |
|  | 0.47 | 2 | 2 | 2 |
|  | 0.8 | 2 | 2 | 2 |
|  | 1.4 | 2 | 2.1 | 2 |
| Grip strength | 0 | 3 | 3 | 3 |
|  | 0.47 | 3 | 3 | 3 |
|  | 0.8 | 3 | 3 | 3 |
|  | 1.4 | 3 | 3 | 3 |
| Gait | 0 | 1 | 1 | 1 |
|  | 0.47 | 1 | 1 | 1 |
|  | 0.8 | 1 | 1 | 1 |
|  | 1.4 | 1 | 1 | 1 |
| Mobility | 0 | 1 | 1 | 1 |
|  | 0.47 | 1 | 1 | 1 |
|  | 0.8 | 1 | 1 | 1 |
|  | 1.4 | 1 | 1.4 | 1 |
| Arousal | 0 | 1 | 1 | 1 |
|  | 0.47 | 1 | 1 | 1 |
|  | 0.8 | 1 | 1 | 1 |
|  | 1.4 | 1 | 1.1 | 1 |
| Exophthalmus | 0 | 1 | 1 | 1 |
|  | 0.47 | 1 | 1 | 1 |
|  | 0.8 | 1 | 1 | 1 |
|  | 1.4 | 1 | 1 | 1 |

## Table S4. Average Number of Rearing, Defecation and Urination and Mean Distance of Hind Limb Splay during Observation Period.

| **Categorical Endpoint** | **Exposure level (MI)** | **Average number during observation period** | | |
| --- | --- | --- | --- | --- |
|  |  | Day -2/-1 prior to dosing | Day 3 | Day 7 |
|  |  |  |  |  |
| Rearing | 0 | 7.2±3.8 | 4.9±3.8 | 6.7±5.6 |
|  | 0.47 | 7.7±3.7 | 4.8±2.0 | 7.4±3.8 |
|  | 0.8 | 7.7±5.8 | 5.6±5.7 | 7.9±5.1 |
|  | 1.4 | 7.8±3.6 | 4.7±4.1 | 8.2±4.4 |
| Defecation | 0 | 0.1±0.3 | 0.0±0.0 | 0.1±0.3 |
|  | 0.47 | 0.0±0.0 | 0.0±0.0 | 0.1±0.3 |
|  | 0.8 | 0.1±0.3 | 0.1±0.3 | 0.3±0.7 |
|  | 1.4 | 0.0±0.0 | 0.0±0.0 | 0.0±0.0 |
| Urination | 0 | 0.1±0.3 | 0.0±0.0 | 0.2±0.4 |
|  | 0.47 | 0.3±0.5 | 0.1±0.3 | 0.5±0.5 |
|  | 0.8 | 0.3±0.5 | 0.3±0.5 | 0.1±0.3 |
|  | 1.4 | 0.3±0.5 | 0.2±0.4 | 0.5±0.7 |
| Distance of hind leg splay | 0 | 58.756±16.456 | 59.492±14.742 | 63.966±21.665 |
|  | 0.47 | 63.688±15.140 | 60.208±11.898 | 60.316±15.309 |
|  | 0.8 | 58.609±18.486 | 68.762±17.652 | 60.922±10.865 |
|  | 1.4 | 56.153±9.540 | 52.514±9.898 | 51.027±12.807 |

## Table S5. Summary of serum haematology and biochemistry.

| **Exposure level (MI)** | **0** | **0.47** | **0.8** | **1.4** |
| --- | --- | --- | --- | --- |
| **Biochemistry** | |  |  |  |
| ALP(U/L) | 150±30.9 | 175.1±36.8 | 151.7±33.9 | 139.5±35.2 |
| ALT(U/L) | 35.4±5.2 | 37.1±6.4 | 35.3±8.1 | 33.7±7.8 |
| AST(U/L) | 106.7±23.4 | 112.4±15.5 | 105±29.6 | 97.4±7.7 |
| r-GT(U/L) | 1.042±0.545 | 1.247±0.462 | 1.325±0.282 | 1.171±0.512 |
| GLU(mg/dL) | 202.7±46.6 | 182.5±30.6 | 225.4±28.8 | 206.7±32.9 |
| D-BIL(mg/dL) | 0.014±0.005 | 0.014±0.005 | 0.013±0.005 | 0.011±0.003 |
| CRE(mg/dL) | 0.48±0.04 | 0.47±0.08 | 0.47±0.07 | 0.49±0.09 |
| BUN(mg/dL) | 15.52±1.23 | 15.35±1.37 | 15.29±1.07 | 14.71±1.63 |
| TP(g/dL) | 6.22±0.27 | 6.2±0.34 | 6.23±0.24 | 6.33±0.22 |
| ALB(g/dL) | 3.33±0.11 | 3.29±0.1 | 3.27±0.07 | 3.27±0.16 |
| A/G() | 1.15±0.05 | 1.13±0.08 | 1.1±0.07 | 1.06±0.1 |
| Na(mEq/L) | 147.4±1.3 | 147±2 | 147.6±1.4 | 148.7±1.9 |
| K(mEq/L) | 6.58±0.73 | 6.92±1.02 | 6.84±0.97 | 6.49±0.87 |
| Cl(mEq/L) | 99.7±0.7 | 99.1±1.1 | 98.8±0.8 | 98.8±1.8 |
| Ca(mg/dL) | 11.94±0.43 | 11.84±0.3 | 12.29±0.35 | 12.14±0.52 |
| P(mg/dL) | 11.5±1.06 | 11.16±0.74 | 11.57±0.51 | 11.6±0.85 |
| CHO(mg/dL) | 57.4±7.7 | 61.1±8.1 | 60.8±14.2 | 70.9±19.8 |
| TG(mg/dL) | 42.2±16.2 | 38.2±14.8 | 46.2±10.4 | 44.4±19.7 |
| LDH(U/L) | 375.4±282.2 | 462.6±177.2 | 355.7±155.3 | 355.1±136.6 |
| CPK(U/L) | 319.6±165.2 | 366.9±97.2 | 317±91.8 | 295±78.9 |
| UA(mg/dL) | 3.65±0.55 | 3.2±0.42 | 3.84±0.64 | 3.74±0.68 |
|  |  |  |  |  |
| **Haematology** | |  |  |  |
| WBCs(103/μL) | 12.828±2.02 | 12.175±2.793 | 12.624±1.988 | 12.345±1.593 |
| RBCs(106/μL) | 8.489±0.482 | 8.447±0.561 | 8.113±0.358 | 8.734±0.437 |
| HGB(g/dL) | 16.67±0.97 | 16.47±1.11 | 15.95±0.51 | 16.88±0.88 |
| HCT(%) | 49.53±2.97 | 49.16±3.56 | 47.6±1.73 | 50.32±2.91 |
| MCV(fL) | 58.36±1.26 | 58.22±1.56 | 58.7±1.63 | 57.62±1.3 |
| MCH(pg) | 19.64±0.46 | 19.51±0.49 | 19.67±0.62 | 19.34±0.64 |
| MCHC(g/dL) | 33.65±0.26 | 33.53±0.43 | 33.53±0.38 | 33.55±0.59 |
| PLT(103/μL) | 948±66.3 | 987.2±89.1 | 904.2±113.3 | 992.2±128.9 |
| NEUT(103/μL) | 1.857±0.511 | 1.659±0.698 | 1.646±0.528 | 1.497±0.367 |
| LYMPH(103/μL) | 10.227±1.918 | 9.781±2.116 | 10.267±1.822 | 10.089±1.699 |
| MONO(103/μL) | 0.289±0.054 | 0.279±0.091 | 0.272±0.066 | 0.33±0.127 |
| EOS(103/μL) | 0.145±0.05 | 0.123±0.024 | 0.13±0.044 | 0.134±0.036 |
| BASO(103/μL) | 0.169±0.057 | 0.155±0.058 | 0.179±0.056 | 0.165±0.029 |
| NEUT(%) | 14.7±3.93 | 13.48±3.59 | 13.07±3.4 | 12.29±3.26 |
| LYMPH(%) | 79.45±4.61 | 80.56±3.77 | 81.17±4.2 | 81.42±4.55 |
| MONO(%) | 2.31±0.61 | 2.3±0.54 | 2.2±0.68 | 2.76±1.2 |
| EOS(%) | 1.14±0.32 | 1.08±0.29 | 1.05±0.36 | 1.07±0.31 |
| BASO(%) | 1.31±0.35 | 1.23±0.25 | 1.42±0.28 | 1.34±0.23 |
|  |  |  |  |  |
| **Coagulation** |  |  |  |  |
| aPTT(second) | 13.54±1.26 | 13.36±1.37 | 13.07±1.19 | 13.4±1.71 |
| PT(second) | 18.32±1.41 | 17.28±1.69 | 16.35±1.93 | 18.18±2.2 |
| Fibrinogen(mg/dL) | 225.31±11.95 | 240.7±15.46 | 236.57±7.84 | **245.29±22.48*** |

*: p < 0.05

## Table S6. Summary of Organ weight.

| **Exposure level (MI)** |  | **0** | **0.47** | **0.8** | **1.4** |
| --- | --- | --- | --- | --- | --- |
| Organ weight(g) | Spleen | 0.6854±0.0675 | 0.6316±0.0831 | 0.6611±0.0993 | 0.6174±0.0724 |
|  | Liver | 11.254±0.976 | 11.177±0.874 | 11.898±0.608 | 11.127±0.579 |
|  | Adrenals | 0.0593±0.0058 | 0.0531±0.0075 | 0.0625±0.0075 | 0.0618±0.0115 |
|  | Kidneys | 2.896±0.256 | 2.931±0.248 | 3.051±0.336 | 2.937±0.224 |
|  | Testes | 3.229±0.191 | 3.195±0.213 | 3.191±0.224 | 3.187±0.196 |
|  | Thymus | 0.4562±0.06 | 0.4519±0.0866 | 0.4525±0.0894 | 0.4192±0.0971 |
|  | Heart | 1.312±0.088 | 1.279±0.067 | 1.3±0.098 | 1.251±0.05 |


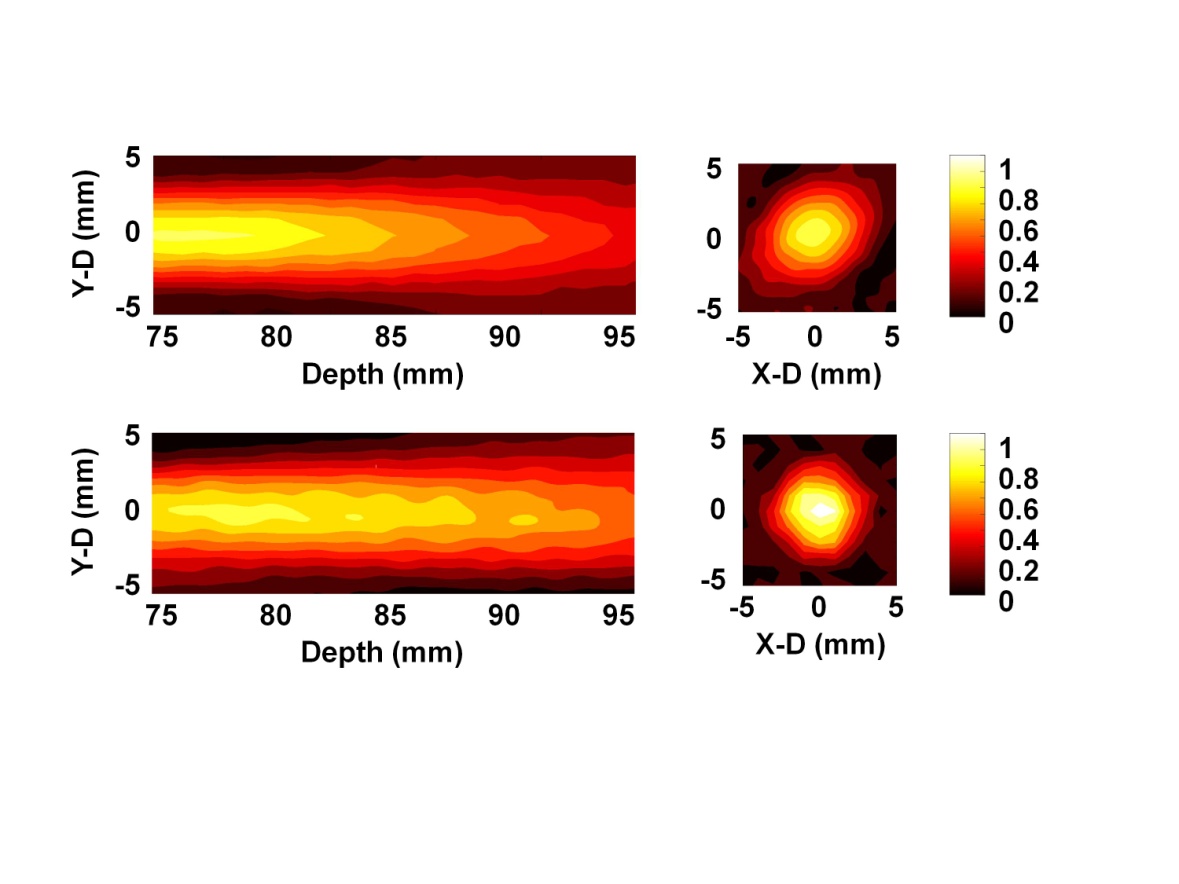


**Figure S1. Pressure measurement of the focused ultrasound transducer.** (a) Free-field measurement. (b) Transcranial measurement when inserting a rat temporal bone skull piece in the beam passage (at depth of 70 mm). The pressure loss when inserting the bone piece was measured to be approximately 10%.

**Figure S2. Confirmation of apoptotic response on neuron cells.** Double staining of Synaptophysin and Annexin V at FUS exposure area in the excessive MB concentration (0.4 ml/kg), heavy FUS exposure (0.8 MI) group after 3 exposures (Day 5).
